# Integrated whole transcriptome and small RNA analysis revealed multiple regulatory networks in colorectal cancer

**DOI:** 10.1038/s41598-021-93531-y

**Published:** 2021-07-14

**Authors:** Hibah Shaath, Salman M. Toor, Mohamed Abu Nada, Eyad Elkord, Nehad M. Alajez

**Affiliations:** 1 College of Health and Life Sciences, Hamad Bin Khalifa University (HBKU), Qatar Foundation (QF), Doha, Qatar; 2 Translational Cancer and Immunity Center (TCIC), Qatar Biomedical Research Institute (QBRI), Hamad Bin Khalifa University (HBKU), Qatar Foundation (QF), PO Box 34110, Doha, Qatar; 3 Department of Surgery, Hamad Medical Corporation, Doha, Qatar; 4 Biomedical Research Center, School of Science, Engineering and Environment, University of Salford, Manchester, UK

**Keywords:** Molecular biology, Cancer, Colorectal cancer

## Abstract

Colorectal cancer (CRC) remains a global disease burden and a leading cause of cancer related deaths worldwide. The identification of aberrantly expressed messenger RNA (mRNA), long non-coding RNA (lncRNA), and microRNA (miRNA), and the resulting molecular interactions and signaling networks is essential for better understanding of CRC, identification of novel diagnostic biomarkers and potential development of therapeutic interventions. Herein, we performed microRNA (miRNA) sequencing on fifteen CRC and their non-tumor adjacent tissues and whole transcriptome RNA-Seq on six paired samples from the same cohort and identified alterations in miRNA, mRNA, and lncRNA expression. Computational analyses using Ingenuity Pathway Analysis (IPA) identified multiple activated signaling networks in CRC, including ERBB2, RABL6, FOXM1, and NFKB networks, while functional annotation highlighted activation of cell proliferation and migration as the hallmark of CRC. IPA in combination with in silico prediction algorithms and experimentally validated databases gave insight into the complex associations and interactions between downregulated miRNAs and upregulated mRNAs in CRC and vice versa. Additionally, potential interaction between differentially expressed lncRNAs such as *H19, SNHG5,* and *GATA2-AS1* with multiple miRNAs has been revealed. Taken together, our data provides thorough analysis of dysregulated protein-coding and non-coding RNAs in CRC highlighting numerous associations and regulatory networks thus providing better understanding of CRC.

## Introduction

CRC is one of the most prevalent types of cancer worldwide^1,2^, with an estimated 9% and 8% of all diagnosed cancers in males and females respectively, to be attributed to CRC in the United States^1^. Better understanding of CRC biology and the development of new strategies and therapies targeting CRC is crucial, as the current traditional options remain limited to chemotherapy, radiotherapy and surgery, which can be highly invasive, involving the use of highly toxic drugs with many undesirable side effects^3,4^. In recent years, scientists are realizing the importance of post-transcriptional regulation and their effects on cellular processes, and in particular, the roles they play in cancer onset and progression. Studying the changes in expression levels of mRNAs, miRNAs, and lncRNAs has become of major importance, as well as understanding the effects of their aberrant expression patterns on downstream effectors and alterations in important regulatory pathways.

Multiple molecular alterations and genetic signatures have been associated with the onset and progression of CRC, which can serve as potential prognostic and predictive biomarkers for the identification of high-risk patients. Of the most common gene signatures associated with CRC is the mutation of *Adenomatous polyposis coli (APC)* gene. The encoded tumor suppressor, in addition to preventing uncontrolled growth of cancerous tumors, regulates beta-catenin, affecting its interactions with E-cadherin responsible for cell adhesion^5^. *Apc*-mutant cells were recently found to be enriched for transcripts encoding several secreted WNT antagonists such as *notum*. The inhibition of NOTUM in *Apc*-mutant cells prevented their expansion and ability to form intestinal adenomas, therefore identifying *notum* expression as a key mediator in early mutation fixation in CRC^6^.

MiRNAs are a large and diverse class of non-coding RNAs, around 18–24 nucleotides in length that are known to play a role in cell proliferation, migration and invasion, and have been suggested as potential strategy to overcome CRC chemo-resistance in vitro and in experimental animals^7,8^. Abnormal expression patterns of miRNAs, detected through microarrays, and more recently, next-generation sequencing, have been implicated in a number of human cancers including breast cancer^9,10^, cervical squamous cell carcinoma^11^, bladder cancer^12^, and melanoma^13^. We previously reported miRNA-320, downregulated in primary CRC, to suppress CRC by targeting SOX4, FOXM1, and FOXQ1. Upon lentiviral mediated re-expression of miR-320, growth and migration of CRC cells in vitro was inhibited, sensitizing them to 5-Fluorouracil (5-FU) therapy, and inhibited tumor formation in SCID mice^14^.

MiRNAs in cancer seldom work alone, and extensive research has provided us with known associations with lncRNAs, another group of non-coding RNAs of around 200 nucleotides^15^, working independently or together as part of an axis in governing specific cellular processes in cancer^16^. As an example, a recent study by Li et al., demonstrates the suppression of CRC cell growth upon knockdown of lncRNA XIST, via regulating microRNA-338-3p/PAX5 axis. This observation may serve as a potential target for the prevention and treatment of CRC^16^. XIST has also been described, in an earlier study, to inhibit 5-FU -induced cell cytotoxicity through promoting thymidylate synthase expression, suggesting the silencing of XIST to be beneficial in overcoming chemo-resistance during of CRC therapy^17^.

Our previous genome-wide mRNA and miRNA expression profiling revealed multiple regulatory networks in CRC including Wnt (wingless-type MMTV integration site family member), matrix metalloproteinase, and TGF-β pathways. Pharmacological inhibition of these pathways led to dose- and time-dependent inhibition of CRC cell growth^18^. In another study, we revealed a number of systemic alterations in gene expression in circulation (peripheral blood mononuclear cells (PBMCs)), compared to the tumor microenvironment of CRC patients, which revealed activation of immune cell trafficking and cellular movement, and suppression of cellular processes related to cell death^19^.

In current study, we performed total RNA-seq and miRNA-Seq on paired CRC and adjacent normal tissue. Computational analyses identified a myriad of affected mRNA, miRNA and lncRNA interactions in CRC, resulting in multiple affected signaling networks. Functional annotation analysis also highlighted activation of cell proliferation and migration as the hallmarks of CRC. More in depth understanding of the roles played by the identified miRNAs and lncRNAs in CRC is crucial in the identification of potential biomarkers.

## Results

### Alterations in mRNA expression in CRC compared to NT

**Figure 1:**
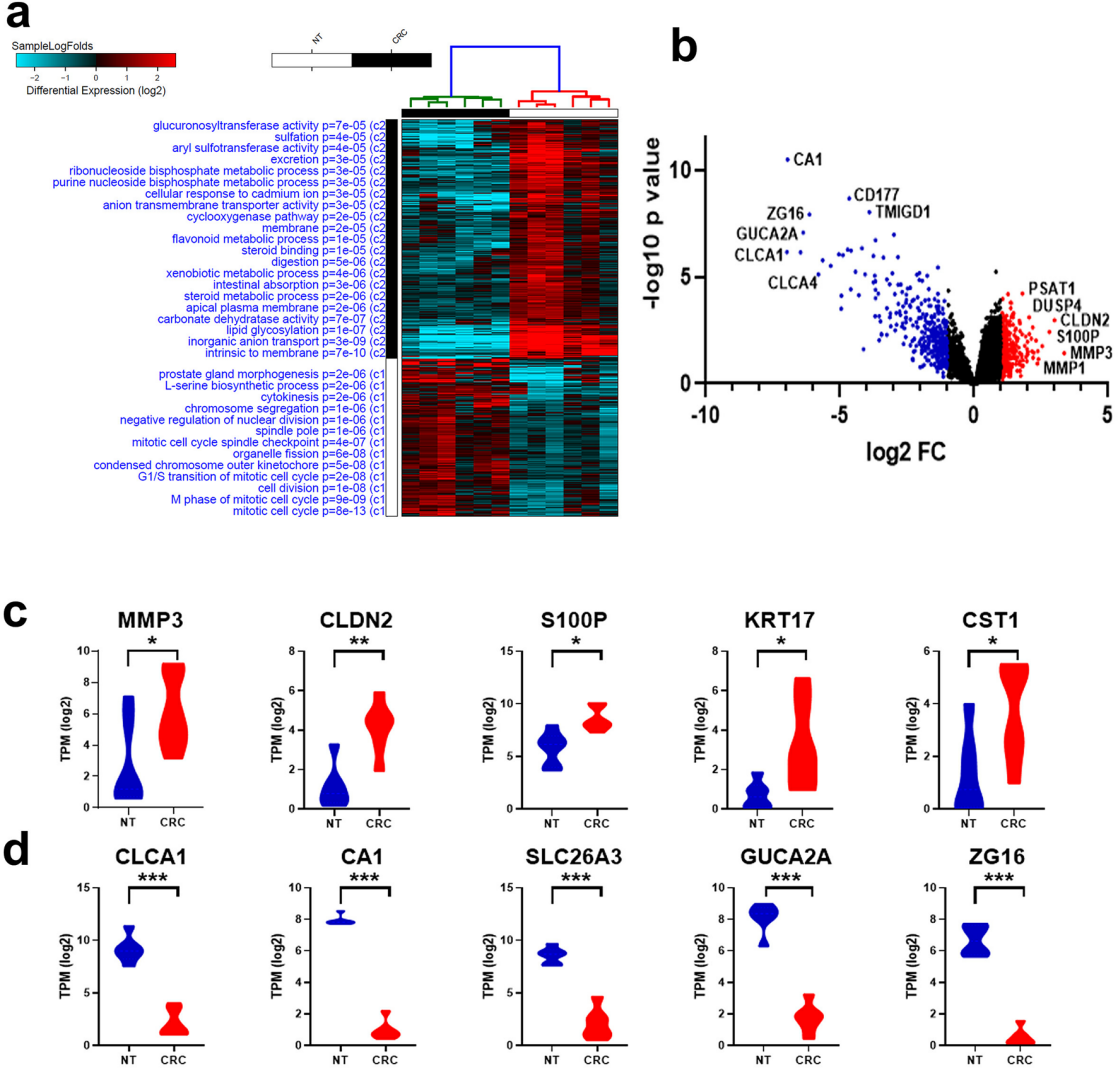
Alteration in mRNA expression in CRC compared to NT. **(a)** Hierarchical clustering of CRC (n = 6) and adjacent normal tissue (n = 6) based on differentially expressed mRNAs between the two groups. Each column represents one sample and each row represents an mRNA. Expression level of each mRNA (log2) in a single sample is depicted according to the color scale. **(b)** Volcano plot representation altered mRNAs in CRC vs. NT. Red and blue colors indicate the genes with significant increased or decreased expression (− 2.0 ≥ FC ≥ 2.0, p < 0.05), respectively, while black color indicates genes with no significant change. Expression of top five upregulated **(c)** or downregulated **(d)** mRNAs in CRC vs NT based on RNA-Seq analysis.

CRC tissue and their adjacent NT from six patients were subjected to total RNA sequencing. Patients’ characteristics and information on tumor site are shown in Table 1. Differential analysis identified several upregulated and downregulated mRNAs in CRC compared to NT (supplementary table 1). Hierarchical clustering of CRC and NT based on differentially expressed (log2) mRNAs between the two groups is illustrated in Fig. 1a. The heat map depicts remarkable differences in the expression levels of several mRNAs (each row representing an mRNA), grouped in relation to their indicated biological processes. Functions related to prostate gland morphogenesis, L-serine biosynthetic process, cytokinesis, chromosomes segregation, and several other cell cycle processes were significantly upregulated (red) in CRC tissue compared to NT, as seen in Fig. 1a. Downregulated functions in CRC tissue compared to NT include inorganic anion transport, lipid glycosylation, steroid metabolic processes and carbonate dehydratase activity (blue). Alterations in mRNA expression between CRC and NT are presented as volcano plot (Fig. 1b), highlighting upregulated mRNAs (red dots) based on log2 FC including PSAT1, DUSP4, CLDN2, S100P, MMP3 and MMP1, among several others. Downregulated mRNAs are presented as blue dots on the volcano plot which include *CA1, CD177, TMIGD1, ZG16, GUCA2A, CLCA1* and *CLCA4*. Figure 1c,d show the top 5 upregulated mRNAs (*MMP3, CLDN2, S100P, KRT17* and *CST1*) and top five downregulated mRNAs (*CLCA1, CA1, SLC26A3, GUCA2A* and *ZG16*) in CRC vs NT based on RNA-Seq analysis (log2 TPM). The top differentially expressed genes identified in our study were compared against the TCGA colon adenocarcinoma (COAD) dataset. Expression of the top 20 differentially expressed mRNAs in our cohort (10 upregulated and 10 downregulated) in the TCGA COAD dataset is shown in supplementary Fig. 1, revealing high concordance with our data.

**Table 1:** Characteristic features of study populations.

Patient no.	Age (years)	Gender	TNM stage	Tumor location	RNA-Seq	miRNA-Seq
1	62	Male	I	Rectosigmoid	No	Yes
2	47	Male	III	Ascending colon	No	Yes
3	65	Female	IV	Sigmoid colon	No	Yes
4	65	Male	II	Cecum	No	Yes
5	65	Male	III	Transverse colon	No	Yes
6	67	Male	III	Descending colon	No	Yes
7	96	Male	II	Sigmoid colon	Yes	Yes
8	59	Female	IV	Ascending colon	Yes	Yes
9	60	Male	II	Rectosigmoid	No	Yes
10	75	Male	III	Rectosigmoid	Yes	Yes
11	65	Male	I	Ascending colon	Yes	Yes
12	52	Male	II	Transverse colon	Yes	Yes
13	69	Male	I	Rectosigmoid	No	Yes
14	60	Female	I	Cecum	No	Yes
15	67	Female	IV	Ascending colon	Yes	Yes

All patients presented with moderately differentiated tumors.

*CRC* colorectal cancer.

### Multiple affected signaling network in CRC compared to NT

**Figure 2:**
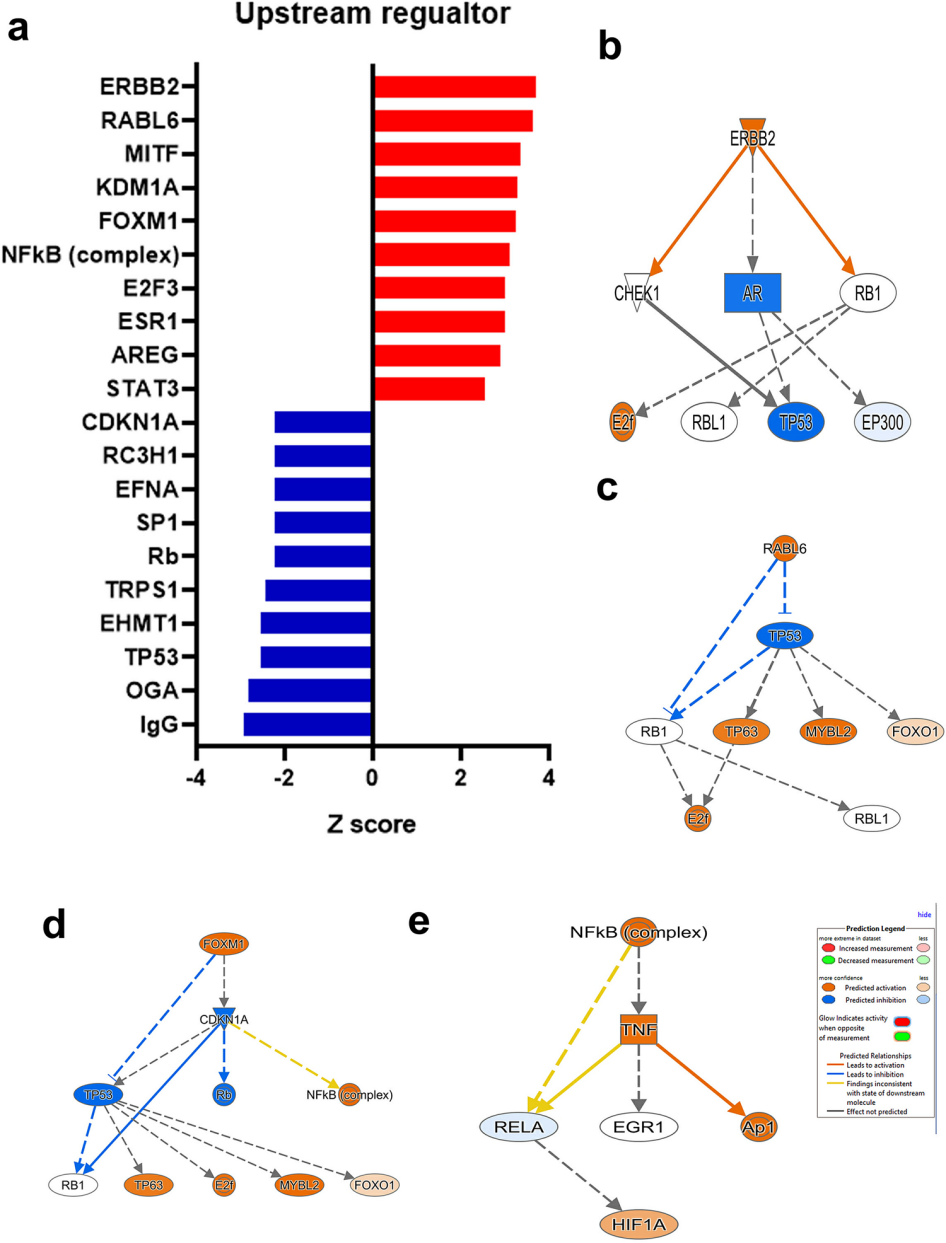
Multiple affected signaling networks in CRC. **(a)** Top altered upstream regulator networks in CRC compared to NT based on IPA analysis. Y-axis indicated the upstream regulator network and the x-axis represent the activation Z score. (**b–e)** are representation of ERBB2, RABL6, FOXM1, and NFKB networks, respectively.

Differentially expressed mRNAs were subsequently subjected to IPA analysis revealing multiple affected signaling networks in CRC compared to NT (Fig. 2a). Those upstream regulators most activated with highest Z scores were ERBB2, RABL6, MITF, KDM1A, FOXM1, NFkB complex, ERF3, ESR1 AREG, and STAT3. Suppressed upstream regulators with negative Z scores were IgG, OGA, TP53, EHMT1, TRPS1, Rb, SP1, EFNA, RC3H1 and CDKN1A. Figure 2b–e show the ERBB2, RABL6, FOXM1, and NFkb complex networks in more detail. ERBB2 is predicted to activate RB1, which in turn activates E2f. ERBB2’s activation of CHEK1, along with its inhibition of AR, simultaneously inhibit TP53 (Fig. 2b). The activation of RABL6 is also predicted to have an inhibitory effect on TP53, with both molecules also inhibiting RB1. The inhibition of TP53 however, leads to TP63, MYBL2 and FOX01 activation, with RB1 and TP63 both activating E2f as a third level downstream effect of RABl6 activation (Fig. 2c). FOXM1 activation is predicted to lead to the inhibition of the CDKN1A network, which inhibits Rb but activates NFkB complex (inconsistent) (Fig. 2d). CDKN1A also inhibits TP53, which has its own downstream consequences as seen in Fig. 2c. Figure 2e highlights the downstream effects of NFkB activation, which activates TNF, which in turn activates Ap1 but suppresses RELA (inconsistent). This inhibition of RELA is predicted to activate HIF1A as an indirect effect of NFkb activation.

### Functional category annotation analysis highlights activation of cell proliferation and migration as the hallmark of CRC

**Figure 3:**
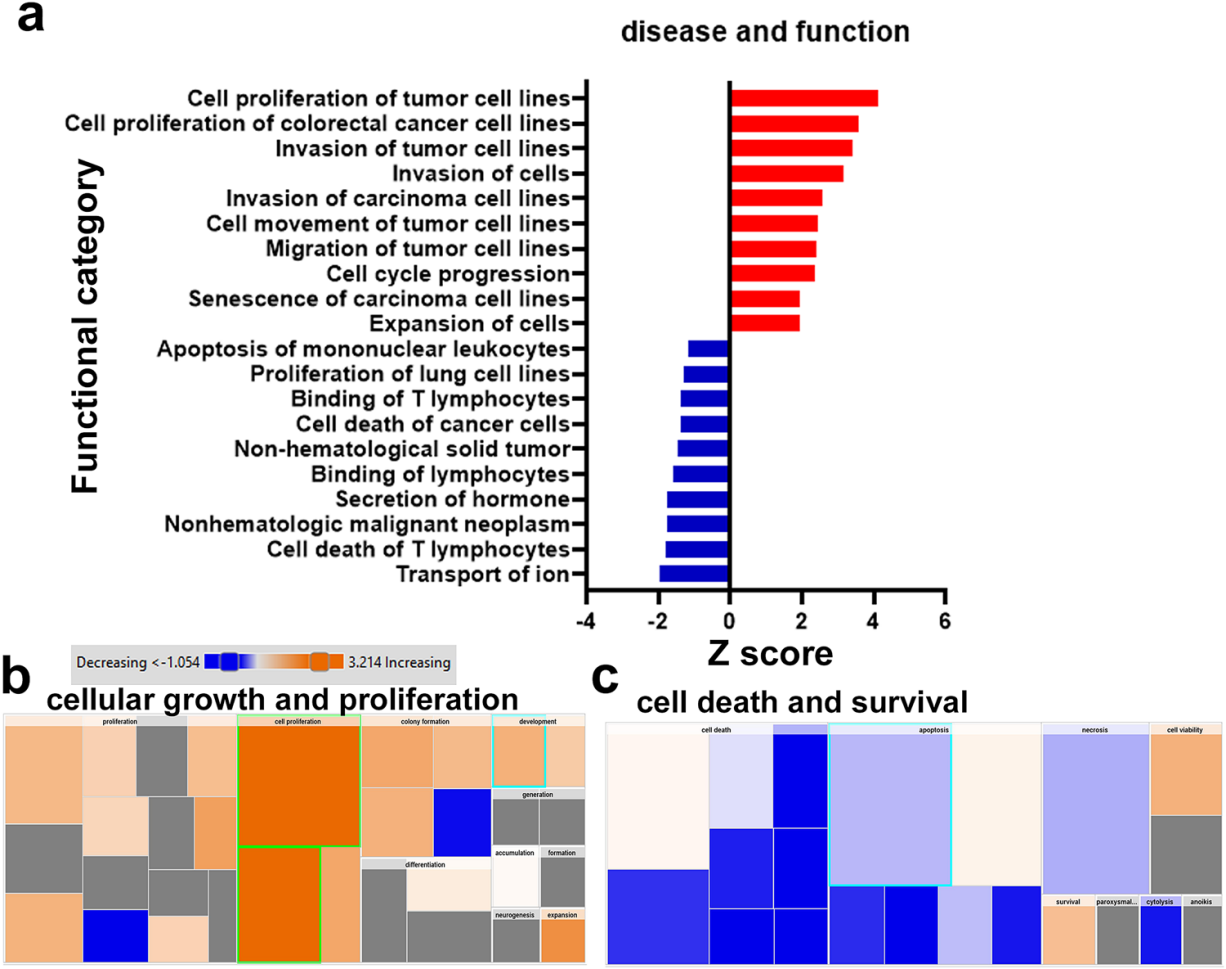
Functional category annotation analysis highlights activation of cell proliferation and migration as the hallmark of CRC. **(a)** Disease and function analysis of differentially expressed mRNAs in CRC. Y-axis indicated the functional category and the x-axis represent the activation Z score. Heatmap representation of cellular growth and proliferation **(b)** and cell death and survival **(c)** functional categories. Activation Z score is depicted according to the color scale.

Functional category annotation of differentially expressed mRNAs brought forward disease and functions predicted to be altered in CRC. Activated disease and functional categories with higher Z scores include tumor cell proliferation, invasion, movement, and cell cycle progression. Those suppressed disease functions include transport of ions, cell death of T lymphocytes, non-hematologic malignant neoplasm, secretion of hormones, binding of T lymphocytes and cell death of cancer cells (Fig. 3a). Figure 3b and c show heat maps illustrating affected downstream biological processes including cellular growth and proliferation, and cell death and survival, respectively. Orange boxes depict activation function whereas blue highlights suppressed functions. As expected in cancer cells, functional categories associated with cellular growth and proliferation, colony formation and expansion are activated whereas those associated with cell death, apoptosis, and necrosis are suppressed, characteristic of cancer cell persistence.

### Alterations in lncRNA expression in CRC compared to NT

**Figure 4:**
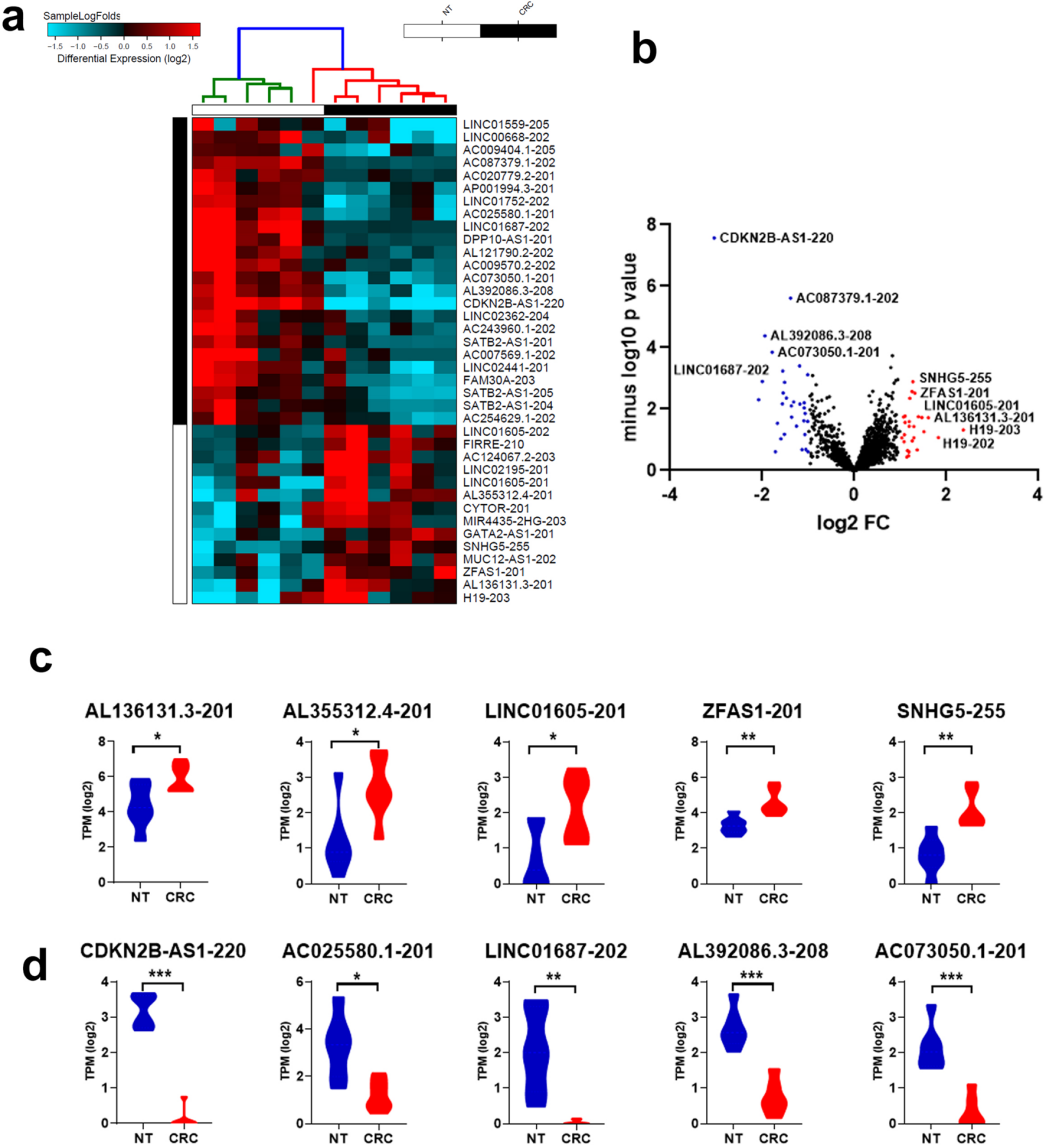
Alteration in lncRNA expression in CRC *vs.* NT. **(a)** Hierarchical clustering of CRC (n = 6) and adjacent normal tissue (n = 6) based on differentially expressed lncRNA transcripts between the two groups. Each column represents one sample and each row represents single lncRNA transcript. Expression levels of each transcript (log2) in a single sample is depicted according to the color scale. **(b)** Volcano plot representation of significantly altered lncRNA transcripts in CRC *vs.* NT. Red and blue colors indicate the transcripts with increased or decreased expression, respectively. Black color indicates no significant change. Expression of the top five upregulated **(c)** or downregulated **(d)** lncRNA transcripts in CRC *vs.* NT based on RNA-Seq analysis.

LncRNA transcripts were also analyzed in the same cohort of six CRC and matched NT revealing several differentially expressed lncRNA transcripts (supplementary table 2). Hierarchical clustering of CRC (n = 6) and adjacent NT (n = 6) based on differentially expressed lncRNA transcripts (log2) is shown in Fig. 4a. Upregulated transcripts in CRC tissues (red) include *LINC01605-202, FIRRE-210, AC124067.2–203, LINC0219-201, LINC01605-201, AL355312.4–201, CYTOR-201, MIR4435-2HG-203, GATA2-AS1-201, SNHG5-255, MUC12-AS1-201, MUC12-AS1-202, ZFAS1-201, AL136131.3–201* and *H19-203,* while several other lncRNAs were downregulated (Fig. 4a). Figure 4b is a volcano plot displaying significantly altered lncRNA transcripts in CRC vs. NT. Blue dots represent the most significantly downregulated lncRNA transcripts whereas the red dots depict the most significantly upregulated lncRNA transcripts in CRC vs. NT. Figure 4c and d show the expression of top five upregulated (*AL136131.3–201, AL355312.4–201, LINC01605-201, ZFAS1-201* and *SNHG5-255)* and downregulated (*CDKN2B-AS1-220, AC025580.1–201, LINC01687-202, AL392086.3–208* and *AC073050.1–201)* lncRNAs in CRC compared to NT*.* In agreement with our data, several of the top upregulated and downregulated lncRNAs from our study showed similar expression pattern in the TCGA COAD dataset (supplementary Fig. 2).

### Alterations in miRNA expression in CRC compared to NT

**Figure 5:**
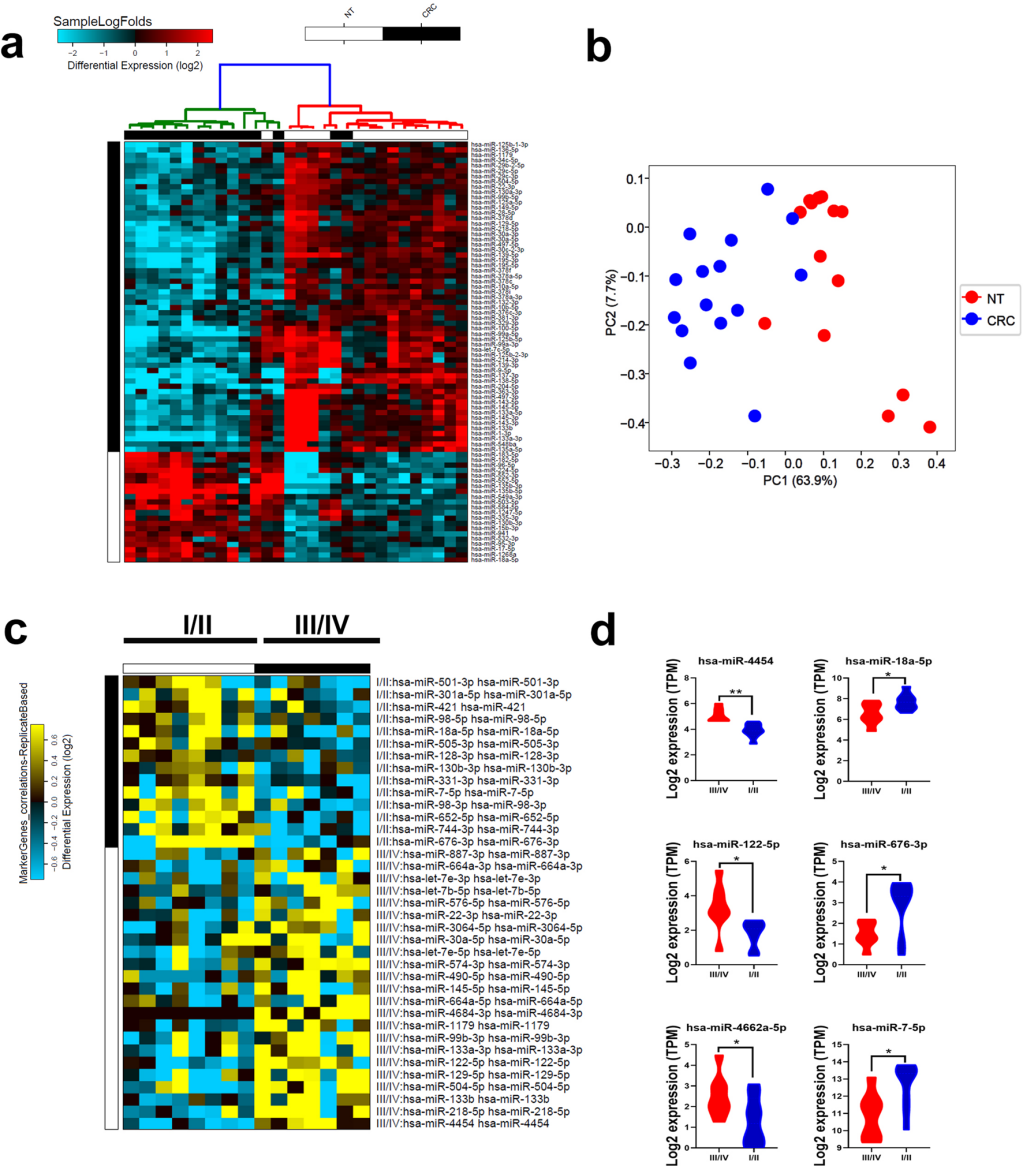
Alteration in miRNA expression in CRC *vs.* NT. **(a)** Hierarchical clustering of CRC (n = 15) and adjacent normal tissue (n = 15) based on differentially expressed miRNAs between the two groups. Each column represents one sample and each row represents a miRNA. Expression level of each miRNA (log2) in a single sample is depicted according to the color scale. **(b)** PCA analysis depicting relatedness of the samples based on PC1 and PC2. **(c)** Heat map illustrating the most differentially expressed miRNAs in CRC in Stage I/II *vs.* Stage III/IV. Expression level in each sample is depicted according to the color scale. **(d)** Expression of three upregulated (*hsa-miR-4454, hsa-miR-122-5p,* and *hsa-miR-4662a-5p*) and three downregulated (*hsa-miR-18a-5p, hsa-miR-676-3p,* and *hsa-miR-7-5p*) in CRC stage III/IV *vs.* stage I/III.

To provide a more comprehensive view of alterations in noncoding RNA transcriptome in CRC, miRNA expression profiling was conducted on 15 CRC tissues compared to the corresponding normal adjacent tissue using miRNA-Seq. Differential expression analysis revealed several differentially expressed miRNAs in CRC compared to NT (supplementary table 3). Hierarchical clustering based on miRNA expression revealed close clustering of CRC away from NT, except for few samples (Fig. 5a). Principle Component Analysis (PCA), showing the degree of relatedness of the samples to each other based on PC1 and PC2, also highlighted the clear segregation of the two groups (NT in red and CRC in blue), with a slight overlap of a few samples as evident in the hierarchical clustering analysis (Fig. 5b). *Hsa-miR-133a-3p, hsa-miR-363-3p, hsa-miR-145-5p, hsa-miR-363-3p,* and *hsa-miR-195-3p*, were among the downregulated while *hsa-miR-135b-5p, hsa-miR-552-5p, hsa-miR-224-5p, hsa-miR-183-5p* and *hsa-miR-552-3p* were among those upregulated in CRC. We subsequently validated top 10 upregulated and top 10 downregulated miRNAs from our study in the TCGA COAD dataset which showed high degree of concordance except for hsa-miR-138-5p, hsa-miR-1-3p, and hsa-miR-9-5p which were not concordant (supplementary Fig. 3). When patients were grouped according to disease stage (stage III/IV *vs.* stage I/II), several miRNAs were found to correlate with disease stage as depicted in Fig. 5c. Among those, *hsa-miR-501-3p, hsa-miR-421, hsa-miR-18a-5p, hsa-miR-128-3p, hsa-miR-7-5p,* and *hsa-miR-744-3p*, were downregulated while *hsa-miR-let-7b-5p, hsa-miR-30a-5p, hsa-miR-145-5p, hsa-miR-1179, hsa-miR-133a-3p, hsa-miR-504-5p,* and *hsa-miR-218-5p* were upregulated in stage III/IV compared to stage I/II. Figure 5d shows the expression of three upregulated (*hsa-miR-4454, hsa-miR-122-5p,* and *hsa-miR-4662a-5p*) and three downregulated *(hsa-miR-18a-5p, hsa-miR-676-3p,* and *hsa-miR-7-5p*) miRNAs in CRC stage III/IV compared to stage I/II.

### miRNA–mRNA–lncRNA computational interaction prediction

**Figure 6:**
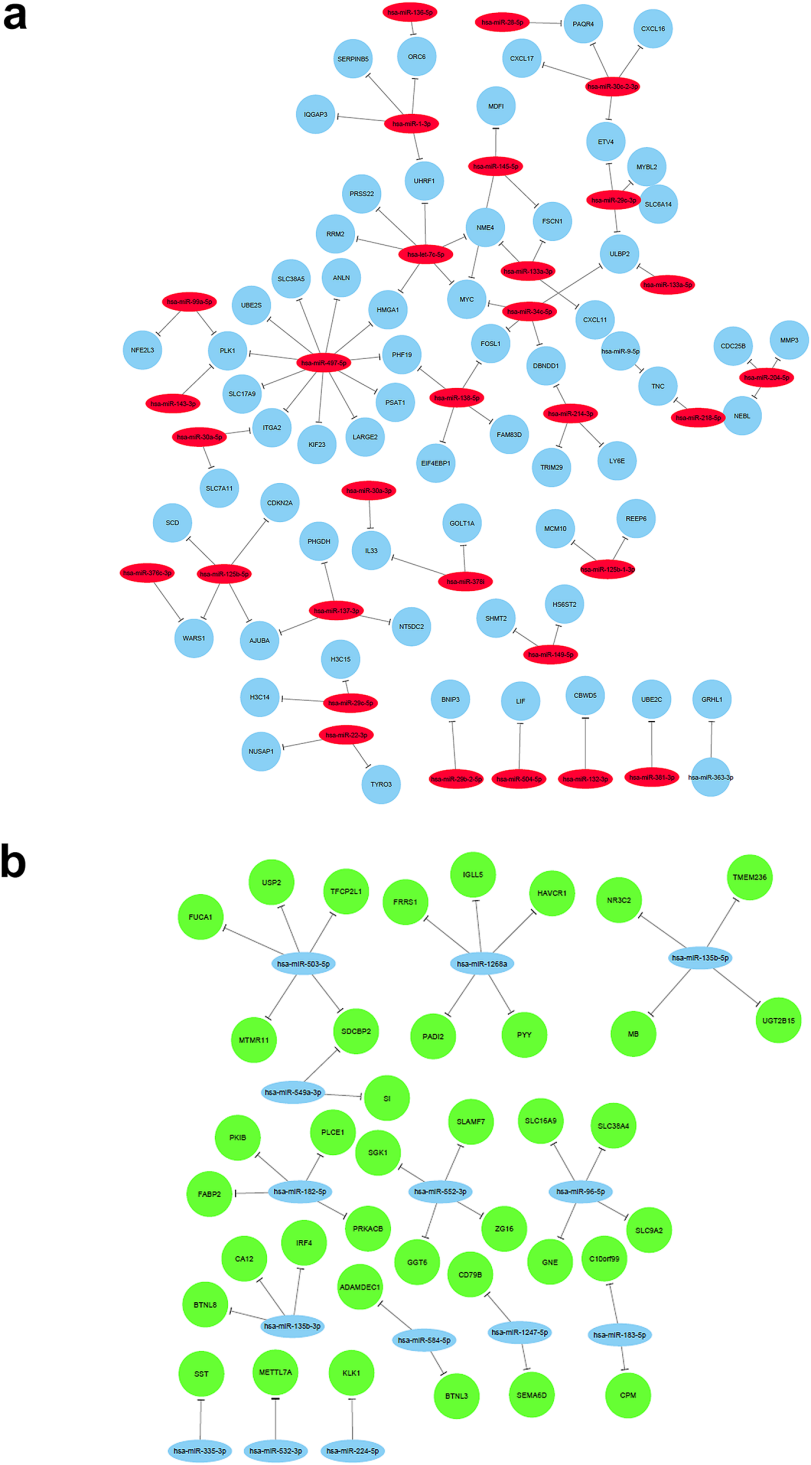
Networks illustrating the interactions between differentially expressed miRNAs and mRNAs in CRC. **(a)** Network illustrating the interaction between downregulated miRNAs and upregulated mRNAs in CRC based on Targetscan prediction and IPA analysis. **(b)** Network illustrating the interaction between upregulated miRNAs and downregulated mRNAs in CRC based on Targetscan prediction and IPA analysis. Only experimentally validated and interactions with high confidence are included in both networks.

We subsequently integrated the miRNA and mRNA expression data using the microRNA filter with IPA thus connecting expression data from the current study with in silico prediction and the IPA manually curated database which identified many regulatory networks (supplementary table 4). We subsequently focused on the differentially expressed miRNAs and mRNAs in CRC and those which were experimentally observed or predicted with high confidence based on IPA analysis which led to the identification of 33 downregulated and 14 upregulated miRNAs and their respected mRNA targets. Visualization of the miRNA-mRNA interactions are depicted using cystoscope (Fig. 6). Starting with hsa-miR-1-3p, the downregulation of this miRNA is predicted to upregulate *IQGAP3, SERPINB5, ORC6* (also associated with the downregulation of *hsa-miR-136-5p*), and *UHRF1*. *UHRF1*, along with *NME4, MYC, HMGA1, RRM2,* and *PRSS22*, interact with the downregulated *hsa-miR-7c-5p* and so on. Figure 6a illustrates in detail, the complex associations and interactions between downregulated miRNAs and upregulated mRNAs in CRC, including several experimentally validated interactions and associations with high confidence according to IPA predictions. Figure 6b shows interactions between upregulated miRNAs and downregulated mRNAs in CRC. For example, upregulated *hsa-miR-1268a* could downregulate *FRRS1, IGLL5, HAVCR1, PYY* and *PADI2*, whereas upregulated *hsa-miR-1356-5p* is predicted to downregulate *NR3C2, TMEM236, MB* and *UGT2B15*. Other mRNAs can be downregulated by more than one miRNA, such as in the case of *SDCBP2*, which is downregulated by the upregulation of both *hsa-miR-503-5p* and *hsa-miR-549a-3p*.

**Figure 7:**
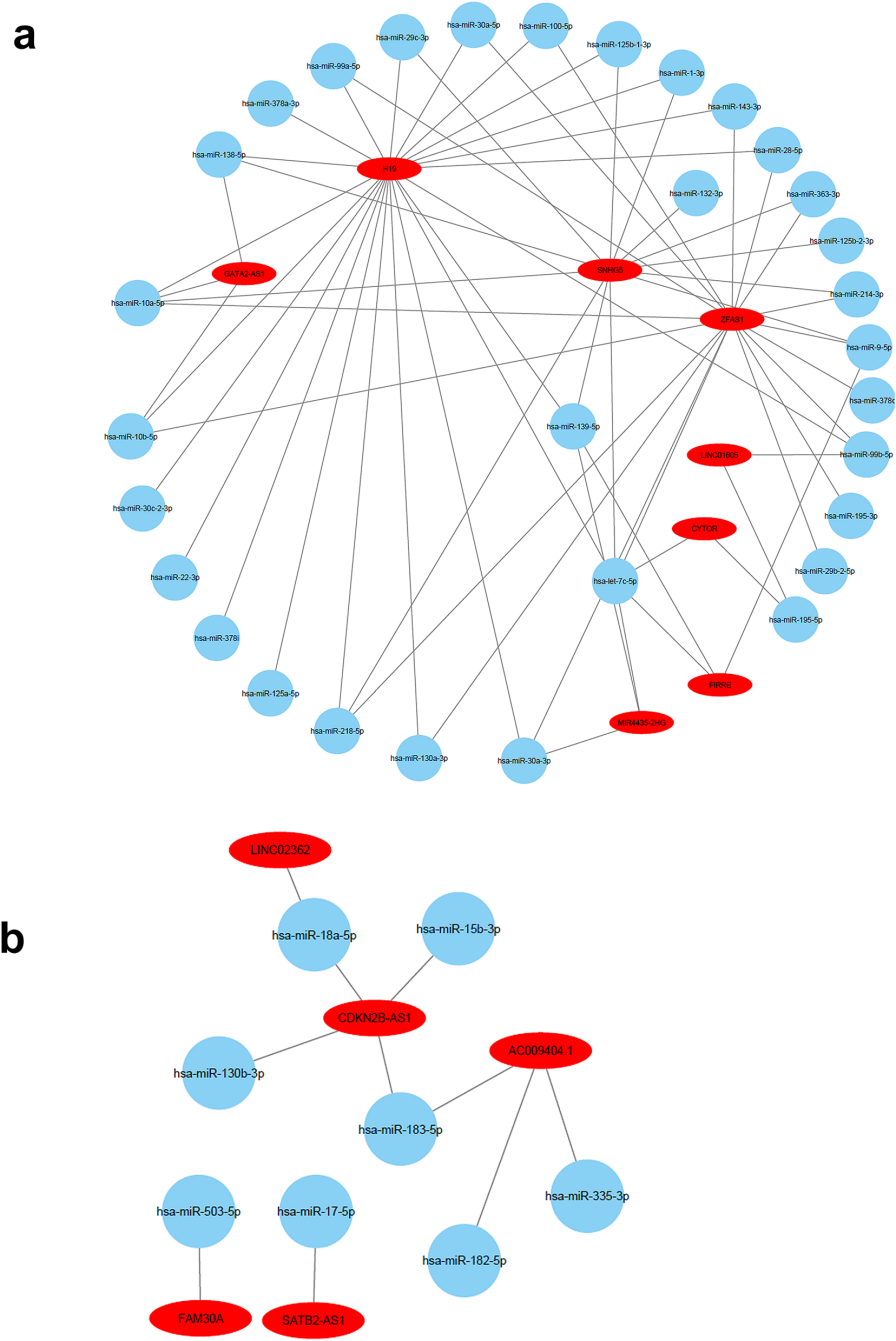
Networks illustrating the interactions between differentially expressed miRNAs and lncRNAs in CRC. **(a)** Network illustrating the interaction between downregulated miRNAs and upregulated lncRNAs in CRC based on DIANA-LncBase v3. 0. **(b)** Network illustrating the interaction between upregulated miRNAs and downregulated lncRNAs in CRC based on DIANA-LncBase v3. 0.

LncRNAs are believed to act as sponge to sequester mature miRNAs and to exert their biological function. Therefore, we explored the DIANA-LncBase database connecting differentially expressed lncRNAs and their corresponding downregulated miRNAs from the current study. For example, Fig. 7a highlights the complex interactions between miRNAs and lncRNAs in CRC in more detail, illustrating the complex associations predicted in CRC. LncRNA *H19* is shown to interact with more than 20 downregulated miRNAs including, *hsa-miR-378a-3p, hsa-miR-30c-2-3p, hsa-miR-22-3p,* and *hsa-miR-125a-5p.* Other miRNAs that interact with *H19*, also interact with other lncRNAs such as *hsa-miR-138-5p*, which interacts with *H19, SNHG5* and *GATA2-AS1*. Inversely, interactions between upregulated miRNAs and downregulated lncRNAs in CRC based on DIANA-LncBase database show *hsa-miR-503-5p* to interact with *FAM30A, hsa-miR-17-5p* to interact with *SATB2-AS1*, and *hsa-miR-18a-5p* to interact with both *LINC02362* and *CDKN2B-AS1*, which in turn, interacts with all of *hsa-miR-15b-3p, hsa-miR-130b-30,* and *hsa-miR-183-5p*. LncRNA *AC009404.1* also shows association with upregulated miRNA *hsa-miR-183-5p*, in addition to both *hsa-miR-182-5p* and *hsa-miR-335-5p* (Fig. 7b).

## Discussion

Colorectal cancer remains one of the most commonly diagnosed cancers worldwide, and a leading cause of cancer related deaths, posing an international burden which is anticipated to worsen according to future predictions^20^. With this knowledge, studies into alternative methods of identifying susceptibility, early diagnosis and treatment is imperative to those affected by CRC. Exploring new avenues through widening our knowledge on the roles played by non-coding RNAs, including miRNA and lncRNA in CRC etiology and progression provides great prospects in improving survival rates of CRC patients. We performed RNA-Seq and computation analyses to characterize the altered mRNA, miRNA and lncRNA expression in CRC compared to NT, as well as identifying the affected signaling networks and predicting the interaction between differentially expressed miRNAs and mRNAs, and miRNAs and lncRNAs in CRC.

The most upregulated mRNAs in CRC according to our cohort includes *MMP3, CLDN2, S100P,* and *KRT17*. In a recent study on colon adenocarcinoma (COAD), the most common type of CRC, Zeng and colleagues looked into transcriptome data from the TCGA COAD dataset and identified MMP3, in addition to several other genes, as prognostic factors for patients with COAD^21^ with functional studies showing improved viability of colon cancer cell lines and decreased apoptosis rate^22^. Other studies have also implicated MMP3 in resistance mechanisms, where expression levels of *MMP3, MMP9* and *MMP14*, as well as some cytokines was increased in drug‐resistant CRC^23^.

Much evidence has also shown the role played by claudin-2 (CLDN2) in breast and CRC^24–27^. Claudins are a family of proteins making up the most important components of tight junctions. Whereas one study shows the upregulation of *CLDN1* and *CLDN2* in CRC, other CLDNs such as *CLDN5, 7, 8*, and *23* were downregulated, indicating a wide range of roles played by these proteins in CRC. Others such as C*LDN11*, *CLDN12*, and *CLDN23* were associated with longer overall survival^27^. The expression of *KRT17* transcript and KRT17 protein as a marker were also significantly associated with poor relapse-free survival (RFS) in stage II CRC^28,29^. Nonetheless, our data highlighted a central role for TP53 in CRC^30^. Signaling networks affected by these alternations in mRNA in CRC include ERBB2, RABL6, FOXM1, and NFKB networks, some of which we have previously reported in breast^31,32^ and CRC^19,32^.

Aside from protein coding RNA transcripts, our data unraveled multiple dysregulated lncRNAs in CRC. Upregulated lncRNA *LINC01605* in our data, otherwise known as *LincDUSP*, has been found to regulate cell cycle genes to resist apoptosis in a study by Forrest et al., where the knockdown of *lincDUSP* in colon tumor cell lines increased the accumulation of cells in early S-phase, indicating increased DNA damage response induction^33^. In a different study, over 80% of CRC samples showed upregulated expression of *LINC01605. Depletion of LINC01605* led to suppression of proliferation, migration and invasion ability of CRC cells in vitro. Furthermore, our data identified LINC01605 to be involved in the miR-3960/SOX11 regulatory axis, which was confirmed experimentally by Hu et al.,^34^. H19 was the most upregulated lncRNA in CRC in our study. Upregulated H19 correlated with poor prognosis and induces epithelial-to-mesenchymal transition (EMT) in CRC. The knock down of hnRNPA2B1, directly bound to H19, attenuated CRC cells ability to migrate and invade^35^. Furthermore, single-nucleotide polymorphisms (SNPs) identified in the promoter region of H19 significantly increased the risk of CRC^36^. H19 lncRNA also acts as a sponge for other miRNAs such as miR-22-3P involved in reduced HDAC2 expression, which strongly promoted CRC lung metastasis^37^. Multiple studies highlight a role for *ZFAS1* in CRC tumorigenesis, where *ZFAS1* silencing reduced CRC cell line migration and invasion ability through miR-34b direct interaction with *ZFAS1* 3' untranslated region (3'UTR)^38^. The ZFAS1/DDX21/POLR1B signaling regulation axis may also be a new biomarker for targeted CRC treatment, where *ZFAS1* knockdown dramatically reduced cancer cell properties and increase apoptosis. This inhibitory effect could be reversed by *DDX21* overexpression^39^. Expanding future studies to include the effects of snoRNAs in CRC is also relevant. LncRNA *ZFAS1* activities extend to rRNA 2'-O-methylation (Me) in CRC initiation and development through oncogenic upregulation of NOP58 and SNORD12C/78 expression in CRC^37^.

Other lncRNAs highlighted in this study are novel transcripts with limited annotations including *AL355312.4–201*, simply referred to as antisense to LRP11. Such lncRNAs provide us with further opportunities to data mine for future studies; exploring their biological roles in CRC as well as their associations and interactions with other mRNAs and miRNAs.

A recent study shows the lncRNA *MCF2L-AS1* to provoke proliferation, invasion and glycolysis of CRC via the crosstalk with miR-874-3p/FOXM1 signaling axis^40^, highlighting the importance of altered interactions between mRNAs, miRNAs and lncRNAs in CRC. In addition to this, lncRNA *SNHG16* was found to promote CRC cell proliferation, migration, and epithelial-mesenchymal transition through miR-124-3p/MCP-1^41^. Our data identified several novel lncRNA transcripts including one of the most significantly upregulated, *SNHG5*, while *SNHG16* was found to be part of a regulatory circuit involving *miR-124-3p*, we found *SNHG5* to interact with *miR-132-3p* (Fig. 7a), which has previously been reported to affect cell proliferation, metastasis and migration of CRC through regulating interaction with CREB5^42^. We identified a number of other different interactions of *SNHG5* with miRNAs including *hsa-let-7c-5p, hsa-miR-363-3p, hsa-miR-125b-2-3p, hsa-miR-214-3p, hsa-miR-9–5-p, hsa-miR-132-3p, hsa-miR-29c-3p,* and *hsa-miR-10a-5p*, amongst others, while other groups have also reported on the significance of *SNHG5* in CRC^42^. Elevated expression of the downregulated *miR-let-7c* in CRC was found to be associated with higher disease control rate (DCR) in metastatic CRC patients treated with anti-EGFR mAbs^43,44^. The upregulation of *miR-let-7c* via the silencing of *Lin28* in another study, promoting apoptosis in CRC cells, indicating the Lin28/let‑7c axis as a potential route for novel therapeutic target in CRC^45^. Let-7c is reported to also function as a metastasis suppressor by targeting MMP11 and PBX3 in CRC, with its downregulation being significantly associated with metastases, advanced TNM stages and poor survival of CRC patients^46^. Our data also records the significant downregulation of lncRNA *CDKN2B-AS1,* which was also found to be aberrantly expressed and is suggested as an optimal diagnostic lncRNA biomarker for COAD by Huang et al.,^47^*. CDKN2B-AS1* has also been confirmed to be downregulated in another study on CRC staging and progression^48^.

Emerging data, including our study, have served to solidify the imperative role played by mRNAs, miRNAs, and lncRNAs, and their resulting interactions within networks and axes as a result of their aberrant expression in CRC. While we have highlighted several aberrantly expressed transcripts described in literature, we have also identified multiple novel transcripts and have therefore identified a number of interactions that warrant further investigation, which could serve as potential biomarkers, giving us a clearer picture on how mRNA and ncRNA play a role in the etiology CRC.

## Materials and methods

### Ethics statement

All patients included in the study were treatment-naïve and provided a written informed consent prior to sample collection. Tumor tissues (TT) and adjacent normal tissues (NT) taken away from tumor margins, were cut from freshly resected tissues by pathologists. The study was performed under ethics committee approvals from Hamad Medical Corporation, Doha, Qatar (Protocol no. MRC-02–18-012) and Qatar Biomedical Research Institute, Doha, Qatar (Protocol no. 2017–006). The characteristics of patients included in this study are provided in Table 1. All experiments were performed in accordance with the ethical principles of the Declaration of Helsinki.

### Sample preparation, RNA extraction, and small RNA library preparation

Colorectal cancer tissue and their non-tumor adjacent tissue specimens were cut into small pieces then snap-frozen and were stored in liquid nitrogen for use in subsequent experiments. Tissues were homogenized using Tissue Homogenization Set from Bioneer Corporation (Daejeon, South Korea) according to manufacturer instructions. Homogenized issue lysates were used to extract RNA using the miRNeasy Mini Kit- QIAGEN (Hilden, Germany) according to manufacturer instructions. In brief, tissue were lysed and kept at room temperature (15–25 °C) for 5 min, chloroform is added, shaking vigorously at room temperature for additional 2–3 min. Samples were centrifuged for 15 min at 12,000×*g* at 4 °C. The aqueous phase was washed with 100% ethanol and added into an RNeasy Mini column in a 2 ml collection tube. RWT and RPE buffers are added, then finally RNA is eluted in water in a fresh tube. The concentration and purity of extracted RNA were measured using NanoDrop 2000c (Thermo Scientific, Waltham, MS, USA). The quantity and quality of extracted RNA was checked using the Agilent RNA 6000 Nano Kit (Agilent Technologies, Santa Clara, CA, USA) and the Agilent 2100 Bioanalyzer (Agilent Technologies, USA) as per the manufacturer’s instructions. Samples with RNA Integrity Number (RIN) > 9 were used for subsequent analyses.

### TruSeq stranded total RNA library preparation

The TruSeq Stranded Total RNA Library Prep Gold kit (Cat #: 20020598) from Illumina was used for total RNA library preparation following the manufacturer’s protocol. Briefly, 100 ng of total RNA extracted from TT and NT samples was subjected to rRNA depletion and then to fragmentation. The first-strand cDNA synthesis was performed with random hexamers and SuperScript II Reverse Transcriptase (Cat#: 18064014) from ThermoFisher Scientific. The second cDNA strand synthesis was performed by substitution of dTTP with dUTP. The double-stranded cDNA is then end-repaired and adenylated. Barcoded DNA adapters were ligated to both ends of the double-stranded cDNA and then amplified. The libraries quality was checked on an Agilent 2100 Bioanalyzer system and quantified using Qubit 2.0 fluorometer (Invitrogen).

### QIAseq small RNA (miRNA) library preparation

For small RNA library preparation, 100 ng of RNA was subjected to 3' ligation followed by 5' ligation and reverse transcription. The next steps included QIAseq miRNA NGS (QMN) bead preparation, cDNA cleanup, and finally library amplification using tube indices, QIAseq miRNA NGS 96 Index IL kit (QIAGEN, Hilden, Germany). The yield of cDNA libraries was quantified using Qubit dsDNA HS assay kit (Invitrogen) and size distribution of the cDNA libraries were determined using the Agilent 2100 Bioanalyzer DNA1000 chip (Agilent Technologies). Libraries were subsequently pooled and were subjected to illumine sequencing at QBRI genomic core.

### Illumina sequencing and bioinformatics analyses

TruSeq Stranded Total RNA libraries were pooled, clustered on a cBot platform, and sequenced on an Illumina HiSeq 4000 at a minimum of 50 million paired end reads (2 × 75 bp) per sample. Similarly, miRNA libraries were pooled and were sequenced using the same platform (5 million reads per sample). Data were subsequently demultiplexed using illumina suggested protocols and the resulting FASTQ files were used for further analysis. For total RNA-Seq analysis, FASTQ files were subsequently pseudo aligned to the Gencode Release 33 index (mRNA and lncRNA) and reads were subsequently counted using KALLISTO 0.42.1 as previously described^10,19,49^. Normalized Transcripts Per Million (TPM) expression values were subsequently subjected to differential expression analysis using 2.0-fold change and < 0.05 p-value cut-off. Transcripts with raw expression values < 1.0 TPM were excluded from the analysis. Hierarchical clustering was performed using cosine for columns and cosine for rows as described before^10,19,50^. Volcano plots was used to illustrate most differentially expressed genes (log two fold change) vs − log10 p value.

For microRNA analysis, FASTQ files were subjected to small-RNA quantification and differential expression analysis in CLC genomics workbench 20.2 using built-in workflow. Initially, raw expression data were mapped to the miRbase v22 reference genome. Read counts of mature miRNA were then normalized using the trimmed mean of M values (TMM) normalization method. Mature miRNA expression values were then subjected to differential analysis as described above. Low abundance miRNAs with expression values < 5.0 count per million (CPM) were excluded from the analysis. The microRNA Target Filter in IPA (using the default database) was subsequently employed to identify miRNA-mRNA networks based on differentially expressed mRNAs and miRNAs from current study. This bioinformatics approach provides insights into the biological effects of microRNAs, using corresponding mRNA expression data as well as experimentally validated interactions from several databases, and the predicted microRNA-mRNA interactions from TargetScan. Only highly predicted and experimentally validated interactions were included in the final analysis. DIANA-LncBase v3 was utilized to identify miRNA-lncRNA interactions. Cytoscape 3.8.1 was used to construct miRNA-mRNA and miRNA-lncRNA networks. Expression values from whole transcriptome RNA-Seq and miRNA-Seq are provided as supplementary tables 5 and 6, respectively.

## Supplementary Information

Supplementary Figure S1.

Supplementary Figure S2.

Supplementary Figure S3.

Supplementary Table S1.

Supplementary Table S2.

Supplementary Table S3.

Supplementary Table S4.

Supplementary Table S5.

Supplementary Table S6.
