# Supplementary material for: Integrated whole transcriptome and small RNA analysis revealed multiple regulatory networks in colorectal cancer
Source: Sci Rep. 2021 Jul 14;11:14456. doi: 10.1038/s41598-021-93531-y (PMC8280114; doi:10.1038/s41598-021-93531-y)
Supplement: Supplementary file 3 — Supplementary Figure S3. [file 41598_2021_93531_MOESM3_ESM.pdf]

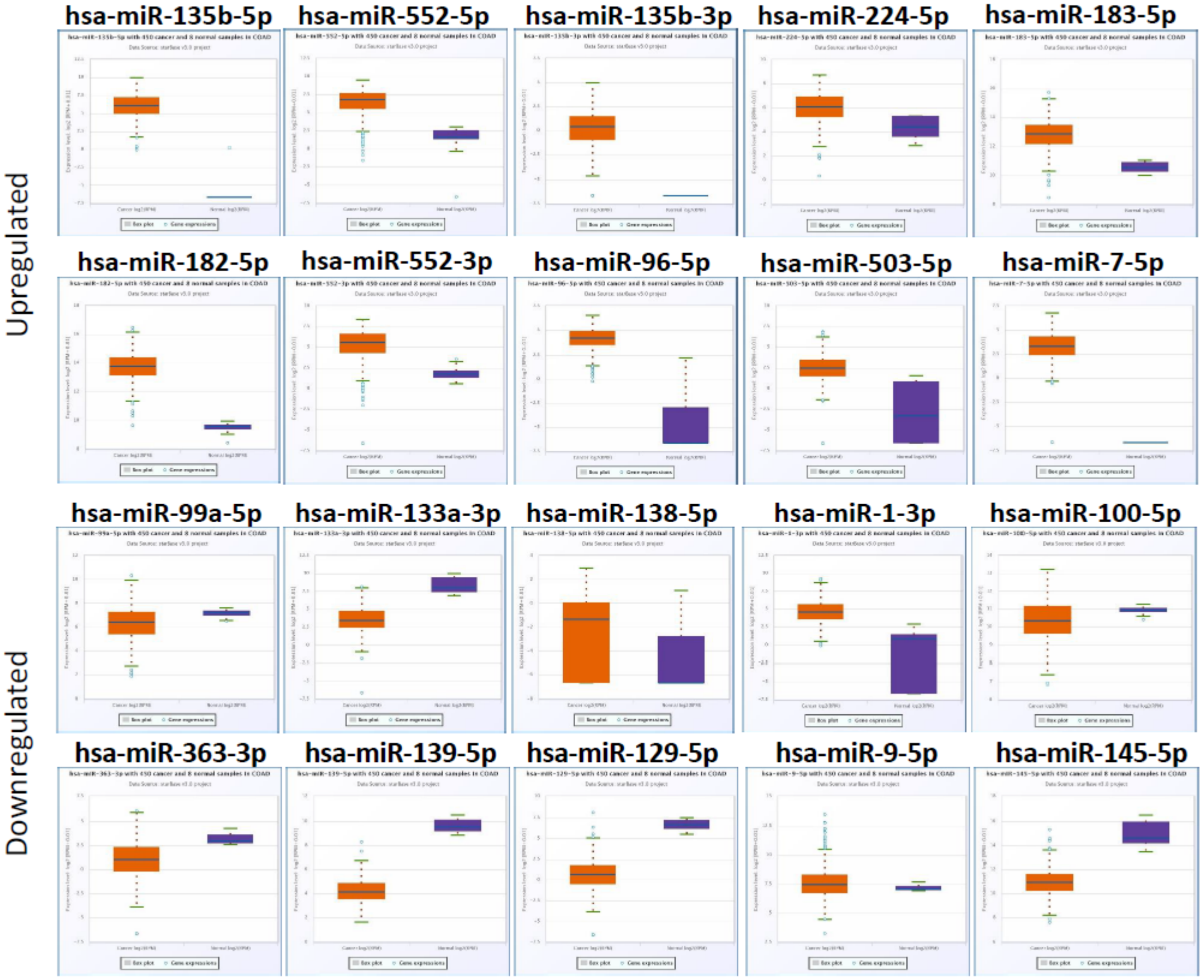

Supplementary figure 3. Expression of top 10 upregulated and top 10 downregulated miRNAs in the TCGA COAS database. Expression of the indicated gene in COAD (n=471) compared to normal tissue (n=41) is shown as box plot. Data were retrieved from the starBase V3.0 database.
